# PeakMatcher facilitates updated *Aedes aegypti* embryonic *cis*-regulatory element map

**DOI:** 10.1186/s41065-021-00172-2

**Published:** 2021-01-28

**Authors:** Ronald J. Nowling, Susanta K. Behura, Marc S. Halfon, Scott J. Emrich, Molly Duman-Scheel

**Affiliations:** 1 Electrical Engineering and Computer Science, Milwaukee School of Engineering, 1025 North Broadway, Milwaukee, WI 53202 USA; 2 Division of Animal Sciences, University of Missouri, Columbia, MO 65211 USA; 3 Department of Biochemistry, State University of New York at Buffalo, NY 14203 Buffalo, USA; 4 Min H. Kao Department of Electrical Engineering and Computer Science, University of Tennessee, Knoxville, 37996 USA; 5 Department of Medical and Molecular Genetics, Indiana University School of Medicine, South Bend, IN 46617 USA; 6 Eck Institute for Global Health, University of Notre Dame, Notre Dame, IN 46556 USA

**Keywords:** *Aedes aegypti*, AaegL5, Functional genomics, Cis-regulatory elements, FAIRE-Seq

## Abstract

**Background:**

The *Aedes aegypti* mosquito is a threat to human health across the globe. The *A. aegypti* genome was recently re-sequenced and re-assembled. Due to a combination of long-read PacBio and Hi-C sequencing, the AaegL5 assembly is chromosome complete and significantly improves the assembly in key areas such as the M/m sex-determining locus. Release of the updated genome assembly has precipitated the need to reprocess historical functional genomic data sets, including *cis*-regulatory element (CRE) maps that had previously been generated for *A. aegypti.*

**Results:**

We re-processed and re-analyzed the *A. aegypti* whole embryo FAIRE seq data to create an updated embryonic CRE map for the AaegL5 genome. We validated that the new CRE map recapitulates key features of the original AaegL3 CRE map. Further, we built on the improved assembly in the M/m locus to analyze overlaps of open chromatin regions with genes. To support the validation, we created a new method (PeakMatcher) for matching peaks from the same experimental data set across genome assemblies.

**Conclusion:**

Use of PeakMatcher software, which is available publicly under an open-source license, facilitated the release of an updated and validated CRE map, which is available through the NIH GEO. These findings demonstrate that PeakMatcher software will be a useful resource for validation and transferring of previous annotations to updated genome assemblies.

## Introduction

The *Aedes aegypti* mosquito, vector of the viruses responsible for the yellow, dengue, chikungunya, and Zika fevers, is a significant threat to global human health. *A. aegypti* is widespread throughout Africa, the Americas, and Asia, threatening a large fraction of human populations [5].

Although originally sequenced in 2007, a chromosome-complete assembly of the *A. aegypti* genome was produced only recently. Matthews, et al. [18] combined long-read PacBio and chromosome conformation capture (Hi-C) sequencing technologies using a new de novo assembly method recently introduced by Dudchenko, et al. [7] to generate a chromosome-complete assembly (AaegL5) for *A. aegypti*. The updated assembly substantially reduced sequence (by ~ 100 mb) and gene duplication (1463 genes previously annotated as paralogs were collapsed). Accuracy and completeness of gene models were increased, resulting in 915 additional genes with > 80% coverage when compared to the respective orthologs of these genes in *Drosophila melanogaster* and the identification of additional chemosensory receptors. The new assembly also improved the fidelity of the M/m locus which will be important to unraveling the underlying mechanisms of sex determination in *A. aegypti*.

*Cis*-regulatory elements (CREs) and the tissue- and developmental-specific chromatin accessibility patterns associated with these regions control gene expression. Mapping CREs and monitoring differences in the state of the chromatin accessibility to these elements is critical to gaining a full and complete understanding of gene expression. For example, differences in chromatin accessibility are associated with *Plasmodium falciparum* infection [27] and immune response [23] in the malaria vector *Anopheles gambiae*, developmental stage in *D. melanogaster* [20], regulation of silk protein genes in *Bombyx mori* [33, 34].

In 2016, Behura et al. [3] performed FAIRE-Seq (Formaldehyde-Assisted Identification of Regulatory Elements combined with next-generation sequencing) [9] on whole embryos to map CREs in the *A. aegypti* genome. FAIRE-Seq identifies open chromatin regions enriched with CREs [9, 13, 20, 29]. Compared with other techniques, FAIRE-Seq requires relatively small amounts of raw genetic material, demonstrates low technical variability, and is associated with a relatively straightforward experimental protocol [19, 28, 30, 31]. A subset of FAIRE peaks was confirmed in vivo to demonstrate enhancer-like activity in *D. melanogaster* reporter assays [3, 21]. Subsequently, the FAIRE data were used to identify CREs associated with expression of olfactory receptor neurons in *A. aegypti* [21]. Despite the value of the FAIRE-seq data set and the AaegL5 genome assembly to the mosquito research community, the embryonic CRE map which had been generated could not be viewed without reprocessing the FAIRE-seq data set for the updated *A. aegypti* genome assembly.

We reprocessed the raw FAIRE sequencing data to create a de novo annotation of CREs in the AaegL5 assembly. As part of that effort, we developed and open-sourced a new tool, PeakMatcher, to match DNA enrichment assay peaks called from the same sequencing data across different genome assembly versions. We applied PeakMatcher to create a list of corresponding peaks from the AaegL3.4 and AaegL5 CRE maps. Using the peak mapping, we confirmed that 14 of 16 experimentally validated peaks in Behura, et al. [3] and [21] were reconstructed in the latest genome assembly (AaegL5, [18]). Our updated and validated CRE map is publicly available through the NIH GEO (GSE162150). We anticipate that PeakMatcher will be useful for validating DNA enrichment assay (e.g., ChIP-Seq, DNase-Seq, or STARR-Seq) annotations across two genome assemblies.

## Results and discussion

### FAIRE peaks properties consistent across *Aedes aegypti* genome versions

We called 128,307 FAIRE peaks for AaegL5 [18] and compared the peaks against the previously called peaks for AaegL3 [22] from Behura et al. [3]. Peaks were called using the same parameters (extent size of 550, no shifting, *p*-value < 0.01 with no FDR control, and the same estimated mappable genome size used in [3]) to reduce bias in the comparison. 23.1, 41.2, and 34.6% of the AaegL5 peaks were located on chromosomes 1, 2, and 3 respectively, while the remaining 1.1% peaks were located on non-chromosome contigs.

**Fig. 1:**
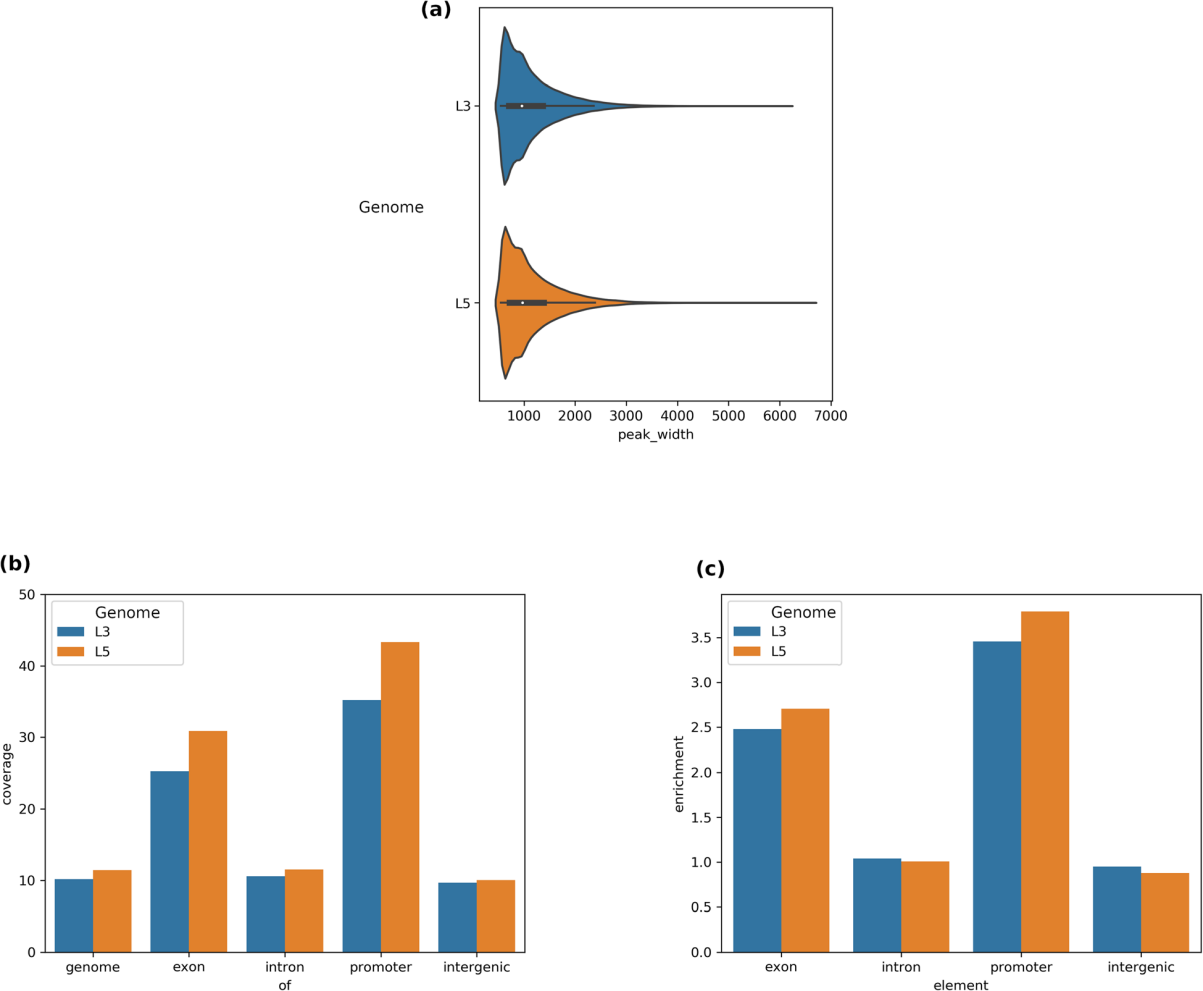
Comparison of FAIRE Peak Characteristics Across the Two *A. aegypti* Genome versions. **a** distributions of peak widths (bp), **b** coverage (percentage of bp) of genomic elements by FAIRE peaks, and (**c**) enrichment of genomic element coverage (multiples of bp) versus expectation assuming uniform distribution of FAIRE peaks

Summary statistics of FAIRE peaks were largely in agreement for both of the genome versions considered (see Table 1). The distributions of FAIRE peaks across AaegL5 and AaegL3 were consistent (see Fig. 1) even though AaegL5 is 106 Mbp smaller, in part because of reduced duplication [18]. We were able to call an additional 6712 FAIRE peaks for AaegL5 that covered an additional 4.8 Mbp of the genome. FAIRE peaks covered an additional 1.2 percentage points (pp) of the fraction of the total genome (see Fig. 1). Increased coverage was observed primarily in exons (5.6 pp) and TSSs (7.9 pp), while coverage of introns (0.9 pp) and intergenic regions (0.3 pp) did not change substantially (see Fig. 1). In turn, enrichments of exons and TSSs were higher in AaegL5 (> 2.5X and > 3.4, respectively; see Fig. 1). FAIRE enrichment of TSSs is consistent with observations in other species [9, 29].

**Table 1:** Genome, Element, and FAIRE Peak Statistics. We calculated the indicated statistics about the two *A. aegypti* genome assemblies, gene sets, and FAIRE peaks

	AaegL3 (Behura, et. al.)	AaegL5 (Us)
**Genome Size**	1401 Mb	1295 Mb
**Gene Coverage (% of bp)**	20.5%	52.4%
**Gene Count**	15,799	14,677
**Gene Width (bp)**	18,198 ± 32,107	46,727 ± 107,809
**Exon Coverage**	2.14%	3.17%
**Exon Count**	71,853	211,311
**Exon Width (bp)**	448 + 558	474 + 705
**Intron Coverage**	18.37%	49.4%
**Intron Count**	49,069	62,581
**Intron Width (bp)**	5182 ± 11,530	10,102 ± 27,245
**Promoter Coverage**^a^	0.56%	0.57%
**Promoter Count**^a^	15,762	14,658
**Mapping Rate**		77.47%
**FAIRE Genome Coverage (Mbp)**	142.9 Mbp	147.6 Mbp
**FAIRE Genome Coverage (%)**	10.2%	11.4%
**FAIRE Peak Count**	121,595	128,307
**FAIRE Peak Width**	1228 ± 562	1138 ± 562

Notes: ^a^We removed promoter windows that extended past the end of a scaffold

### PeakMatcher method

We created a new method and software package called PeakMatcher for matching peaks from the same experiment across different genome assemblies. PeakMatcher is targeted towards cases where the same DNA enrichment assay data is being compared across two assembly versions. BEDTools [25, 26] and similar tools focus on analysis of overlaps of coordinate intervals, but this is not appropriate when two different genome assembly versions have very different coordinate systems. PeakMatcher uses aligned sequencing reads that are common to peaks in both genomes to match the peaks. It is an alternative to approaches based on whole-genome alignments (e.g., using MUMmer [17] or liftOver [12]).

We initially attempted to match peaks across the two *A. aegypti* genome assemblies based on whole-genome alignments, but we were only able to successfully find matches for ~ 40% of the peaks (data not shown). As we did not have access to the original AaegL3 alignments, we re-generated the alignments and re-processed the peaks. We validated the re-processed AaegL3 peaks against the original peak list distributed by Behura et al. [3]. We then matched peaks across the two genome versions using our PeakMatcher method. PeakMatcher was able to match 73.7% of the 124,959 re-processed AaegL3 peaks to 78.9% of the 128,307 AaegL5 peaks. The distributions of matched and all AaegL5 peaks were similar; 22.7, 40.5, and 35.8% of the matched peaks were located on chromosomes 1, 2, and 3 respectively, while the remaining 1.1% peaks were located on non-chromosome contigs.

Methods for combining long read and/or chromosome capture sequencing with next-generation sequencing can generate vastly improved and chromosome-complete assemblies. Fueled by these recent leaps in capability, we anticipate that a number of genomes will be re-sequenced and re-assembled. Functional genomics data sets will need to be reprocessed for use with the reassembled genomes. We anticipate that PeakMatcher will be a useful resource for transfer and validation of previous annotations to updated assemblies.

### Experimentally validated peaks matched across genome versions

Sixteen AaegL3 FAIRE peaks were previously demonstrated to act as enhancers in transgenic insect reporter assays [3, 21]; 14 of these 16 AaegL3 peaks were matched to peaks in AaegL5 by PeakMatcher (see Table 2). Adjacent genes were consistent for 12 of the 14 matched peaks; inconsistencies for at least two of the three remaining peaks were clearly attributable to differences in the assemblies considered here (data not shown).

**Table 2:** FAIRE Peaks Matched Across Aedes Genome Versions. We compiled a list of 16 AaegL3 peaks experimentally validated to have enhancer-like properties using transgenic reporter assays from Behura, et al. [3] and Mysore, et al. [21]. AaegL3 peaks were matched to corresponding AaegL5 peaks using PeakMatcher by finding sequencing reads overlapping both pairs of peaks. Each match peaked was further validated by comparing the local genes in the AaegL3 and AaegL5 assemblies

AaegL3 Peak	Matching AaegL5 Peaks	Nearby Genes Agree
supercont1.2641:1068–1902	2:112926534–112,928,916	Yes
supercont1.381:720103–720,682	2:112926534–112,928,916	Yes
supercont1.551:501192–503,018	3:315077384–315,078,301, 1:3572116–3,573,959	Yes (chromosome 1 peak)
supercont1.174:341062–341,799	No match	
supercont1.237:1279560–1,280,173	3:305593808–305,594,529	Yes
supercont1.16:273854–274,851	3:246347420–246,348,664	Yes
supercont1.160:604315–605,761	1:150742156–150,743,754	Yes
supercont1.440:550819–551,917	1:310314461–310,315,597	Yes
supercont1.128:2089446–2,090,042	2:377442633–377,443,669	Yes
supercont1.911:297903–298,590	2:22088421–22,089,581	No (hex2)
supercont1.635:654750–655,775	2:190755244–190,756,471	Yes
supercont1.1782:38974–39,907	No match	
supercont1.671:130269–131,236	3:53452438–53,454,623	Yes
supercont1.199:700946–702,158	1:121501670–121,502,551	Yes
supercont1.54:975577–976,601	3:368086709–368,088,017	No (onecut)
supercont1.123:863985–864,746	1:220758917–220,759,600	Yes

### FAIRE peak coverage of TSSs associates with gene expression

FAIRE peaks indicate nucleosome-depleted regions associated with regulatory activity. We compared the overlap of FAIRE peaks with 500 bp windows upstream (downstream for negative strand) of transcription start sites with genes indicated to be expressed by RNA-Seq [2]. TSSs for 6611 (65.5%) of the 10,089 genes with increased and 1484 (32.4%) of the 4587 genes with decreased differential expression were overlapped by FAIRE peaks. The differences in frequencies are statistically significant (*p*-value < 10^− 100^, χ^2^ test of independence). We concluded that the FAIRE peaks successfully identify active promoters, consistent with observations in other species [9].

### *M/m* locus

For the first time, a high-quality assembly of the M/m locus is available through the AaegL5 genome of *A. aegypti,* in which a dominant M locus establishes the male sex (male genotype = *M/m;* female genotype = *m/m* [18];). We analyzed the FAIRE peaks in a ~ 1.5 Mb region (151.68–152.95 Mb) in the predicted M/m locus (see Table 3, [18]). Peaks were associated with the TSSs (within 2.5 kb upstream) of five *long non-coding RNA (lncRNA)* genes: *AAEL020975, AAEL022711, AAEL024704, AAEL025015,* and *AAEL026346*. FAIRE elements were also associated with two protein-coding genes*, Nix* and *myo-sex*. *Nix*, which encodes a male-determining factor that is necessary and sufficient to drive male-specific development in *A. aegypti* [11], overalapped with FAIRE peaks located at the TSS and within an intron of this gene. The *myo-sex* gene, which is required for *A. aegypti* male flight [1, 10], is associated with eight intronic FAIRE peaks. FAIRE elements associated with these M/m locus genes (Table 3) may function as CREs that regulate sex-specific gene expression in *A. aegypti*.

**Table 3:** FAIRE Peak-Gene Overlaps in the M/m Locus. We analyzed the FAIRE peaks in a 2.2 Mb region (1:150716898–152,949,239) in the predicted M/m locus. 10 protein-coding and 36 long-noncoding RNA genes were located in the region. For genes with FAIRE peak overlaps, we listed the gene IDs, names (if known), gene types (protein-coding or long noncoding RNA), whether the transcription start site (2.5 Kbp upstream region) was overlapped by a FAIRE peak, and the number of FAIRE peaks overlapping introns of each gene

Gene ID	Gene Name	Gene Type	TSS Overlapped by FAIRE Peaks	Number of Peaks Overlapping Introns
AAEL020975		lncRNA	X	
AAEL021838	*myo-sex*	protein		8
AAEL022711		lncRNA	X	
AAEL022912	*Nix*	protein	X	1
AAEL024704		lncRNA	X	
AAEL025015		lncRNA	X	
AAEL026346		lncRNA	X	

FAIRE-seq mapping of open chromatin associated with the M/m locus represents a first glimpse at regions that could potentially have different chromatin signatures in male and female mosquitoes. The FAIRE DNA sequenced by Behura et al. [3] was prepared from a combination of male and female embryos. It would be interesting to pursue separate FAIRE-seq analyses in male vs. female mosquitoes, particularly once complete sequence information is available for the entire M and m regions. This would permit a more detailed understanding of both genetic, as well as epigenetic, differences between the two sexes. It is also interesting to note the many FAIRE elements associated with lncRNA genes residing at the M/m locus (Table 3). Although the roles of these genes have not yet been described in *A. aegypti,* our detection of open chromatin within and flanking these genes suggests that lncRNAs could play critical roles in the regulation of sex-specific development and differentiation.

## Conclusion

Next-generation sequencing (NGS) drove a massive increase in genome sequencing by lowering costs and increasing per-base calling quality. Unfortunately, many of the resulting assemblies were fragmented. NGS has recently been combined with long-read and chromosome conformation capture (e.g., Hi-C) sequencing to produce new, vastly improved genome assemblies [7]. Resequencing and reassembly of *A. aegypti* demonstrated the power of these techniques, and many genomes will likely be re-sequenced and re-assembled in the next few years, particularly through efforts such as the Earth BioGenome project [14]. The approaches developed here will be useful to other researchers facing similar needs to reprocess functional genomics data for these updated genome assemblies.

We updated whole-embryo *A. aegypti* FAIRE sequencing data for the new AaegL5 genome assembly and validated the reprocessed data to ensure consistency with the previous FAIRE peaks. The resulting FAIRE peak lists are available to the public through the NIH GEO (GSE162150), where they will continue to be available to the insect vector community. Additionally, we anticipate that PeakMatcher will be useful to other researchers who are validating re-annotated DNA enrichment assay (e.g., ChIP-Seq, DNase-Seq, or STARR-Seq) data on updated genome assemblies.

## Methods

### FAIRE peak processing

FAIRE-Sequencing of *A. aegypti* whole embryos was originally performed by Behura, et al. [3]. Raw FAIRE-Seq sequencing data were downloaded from the NIH SRA (SRR2530418, SRR25304189, and SRR25304120). The *A. aegypti* AaegL3.5 [22] and AaegL5.2 [18] genomes and gene sets retrieved from Vectorbase [8]. 382,611,452, 341,380,350, and 296,902,904 sequencing reads were cleaned with trimmomatic (ILLUMINACLIP:TruSeq3-PE.fa:2:30:10 LEADING:3 TRAILING:3 SLIDINGWINDOW:4:15 MINLEN:36) [4]; 340,532,066, 305,899,612, and 274,957,716 paired reads survived cleaning.

The paired reads were aligned to the two reference genomes using BWA backtrack [15], and filtered with SAMtools [16] to remove reads with mapping qualities < 10, unmapped reads, and secondary alignments of reads. 179,608,283, 152,618,302, and 148,862,656 (total of 52.2%) and 171,583,885, 145,715,076, and 142,034,403 (total of 49.9%) of the reads aligned to AaegL3 and AaegL5, respectively, survived filtering. 92.6 and 92.5% of the AaegL3- and AaegL5-aligned reads were properly paired. 57.0 and 56.9% of the AaegL3- and AaegL5-aligned reads were identified as duplicates by Picard tools [24]. (Note that we did not subsequently filter out duplicate reads since MACS already does so as part of its workflow.) Average read lengths were similar across the two sets of alignments; the average lengths were 97, 96, and 97 bp, respectively, for each biological replicate. Insert sizes of 134.6 ± 16.6, 141.2 ± 19.0, and 135.8 ± 16.9 (AaegL3) and 135.0 ± 17.3, 240.5 ± 873.2, and 136.2 ± 17.5 (AaegL5) bp were observed.

Peaks were called from pooled alignments of the three biological replicates using MACS2 [32] (−t rep1.bam rep2.bam rep3.bam --nomodel -p 0.01 --extsize 550 -g 1.24e9). No controls were used. We used the default single-ended mode (BAM) which uses the 5′ end tag, disabled the shifting model (−-nomodel), used a *p*-value cutoff of 0.01 (in place of false discovery rate control), a shift size of 550 bp (−-extsize 500 bp), and an estimated mappable genome size of 1240 Mb to match the parameters used in [3] and facilitate comparison.

### Peak-genomic element overlap enrichment analysis

Gene and exon coordinates were extracted from GFF3 files. Intronic regions were calculated by subtracting exon intervals from gene intervals using BEDtools [25, 26] subtract (default parameters). TSSs were identified as 500 bp windows upstream (positive strand) or downstream (negative strand) of genes using custom Python scripts.

Peak and element intervals were sorted by chromosome and then starting position using “sort -k 1,1 -k2,2n.” Overlaps between FAIRE peaks and genomic elements were determined with “bedtools intersect -a elements.bed -b peaks.bed -f 0.1”. Enrichment in peak-element overlaps was calculated using the following equations:

$$ \mathrm{fraction}\ \mathrm{of}\ \mathrm{peak}\mathrm{s}=\mathrm{observed}\ \mathrm{peak}-\mathrm{element}\ \mathrm{intersection}\ \left(\mathrm{bp}\right)/\mathrm{total}\ \mathrm{peak}\ \mathrm{coverage}\ \left(\mathrm{bp}\right) $$

fraction of genome = total element coverage (bp)/genome size (bp)

$$ \mathrm{enrichment}=\mathrm{fraction}\ \mathrm{of}\ \mathrm{peaks}/\mathrm{fraction}\ \mathrm{of}\ \mathrm{genome} $$

### Matching peaks across genome versions

The PeakMatcher method identifies corresponding peaks called for two different genome assembly versions from the same DNA enrichment assay sequencing data. One genome (usually the older genome which has been previously validated) is defined as the source genome, and the second genome (usually the new genome to be validated) is defined to be the target genome.

**Fig. 2:**
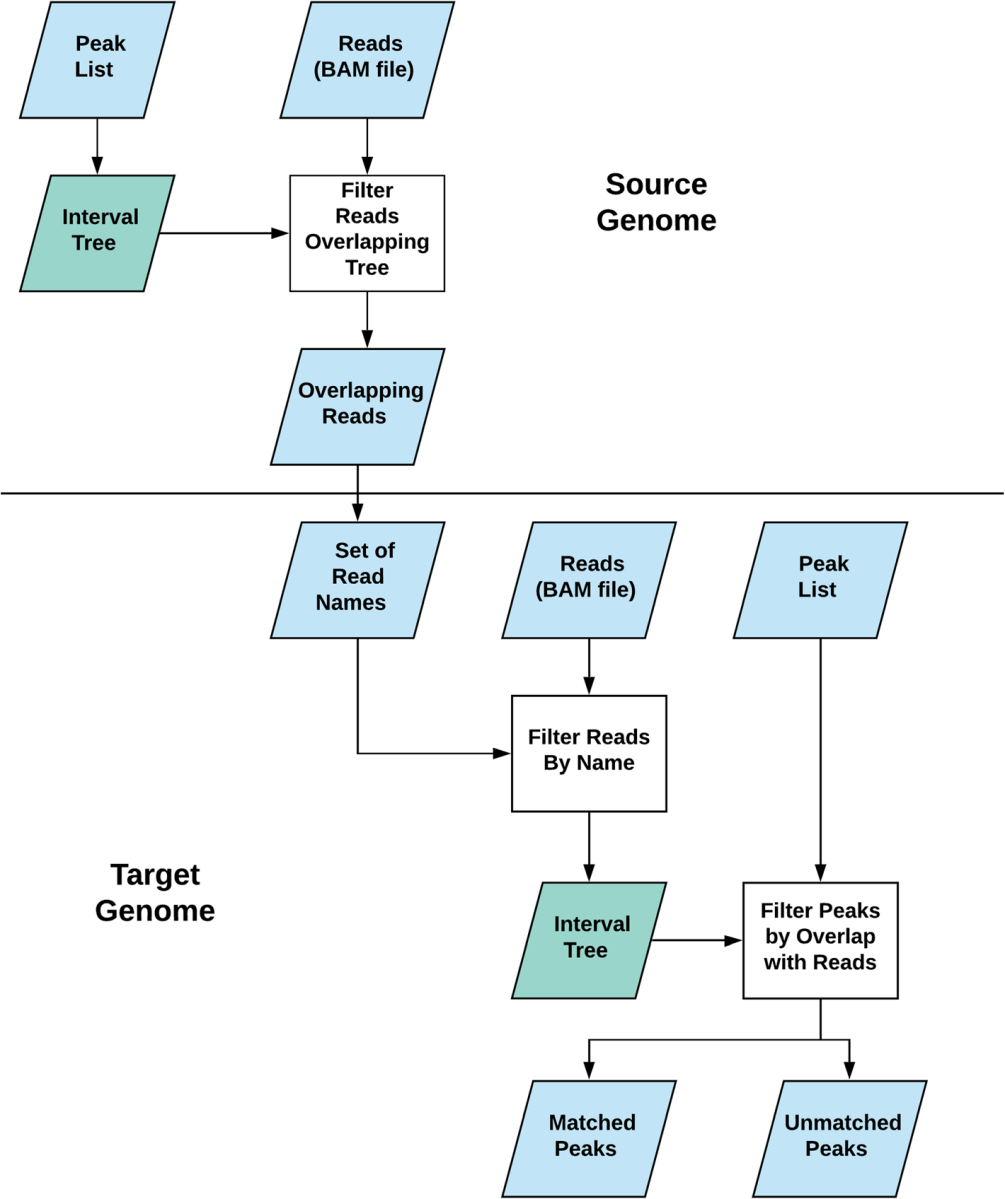
PeakMatcher Workflow. PeakMatcher matches each peak in the source genome to one or more corresponding peaks in the target genome. PeakMatcher finds the sequencing reads that overlap each peak and outputs a table of peak and read names. In the second step, sequencing reads aligned to the target genome are identified for overlapping peaks in the target genome. The mappings of read names to peaks are used to match peaks from the source and target genomes. The final many-to-many table of source and target peaks is output to a text file

PeakMatcher operates in two steps (see Fig. 2). First, peaks from the source genome are associated with aligned reads. PeakMatcher takes the peak lists (e.g., generated by MACS2) and a source genome BAM file as inputs. Each peak is represented as a tuple of four elements (chromosome, start position, end position, peak and name) and an interval tree of peaks is constructed for each molecule (e.g., chromosome, chromosome arm, or scaffold). Interval trees are a specialized type of search tree data structure that enable fast O (log N) overlap queries (e.g., return all intervals in the tree that overlap a given query interval) [6]. Every read in the BAM file is represented as a tuple of three elements (chromosome, start position, and end position) and used to query the tree for overlaps with the called peaks. A list of read-peak names pairs is output.

In the second step, peaks from the source genome are associated with corresponding peaks from the target genome. In the same fashion as the source genome, an interval tree is constructed from the target genome peak list, and the tree is queried to find peaks overlapping each read. A list of target peak-read pairs is generated, and the original list of source peak-read pairs is read in. The two lists are joined on the read names to generate a final list of “matched” peaks as peak-peak pairs.

AaegL3 FAIRE peaks were matched to AaegL5 FAIRE peaks using this method. Sixteen experimentally validated AaegL3 FAIRE peaks listed in Table 2 of Behura, et al. [3] and Table 1 of Mysore, et al. [21] were identified for further validation. The Vectorbase genome browser was used to manually inspect the regions around each peak pair to confirm the match by verifying that neighboring genes in AegL3 for each peak were present in AaegL5.

### RNA-Seq analysis

RNA-Seq data were retrieved from Akbari, et al. [2]. Genes were partitioned into high and low differential expression groups using a FPKM threshold of 1. TSSs for each gene present were extracted as described above. Overlaps between TSSs and peaks were determined using the BEDtools intersect command: “bedtools intersect -a promoters.bed -b peaks.bed -wa -F 0.1 -u”. Statistical differences in promoter overlap counts were confirmed with a Chi-squared test.

### *M/m* locus

We followed the procedure described in the genomic element overlap section above to analyze the *M/m* locus. We restricted our analysis to FAIRE peaks, introns, and TSSs overlapping the *M/m* locus region (1:151.68–152.95 Mb).

## Data Availability

The original FAIRE sequencing data from Behura, et al. [3] is available from the NIH SRA (SRR2530418, SRR25304189, and SRR25304120). We made our re-processed AaegL3 and AaegL5 FAIRE peak lists available through the NIH GEO (GSE162150). The PeakMatcher software is hosted under a separate GitHub repository (https://github.com/rnowling/peak-matcher). The software is licensed under the open-source Apache Software License v2.

## Abbreviations

AaegL3: *A. aegypti* genome assembly version 3
AaegL5: *A. aegypti* genome assembly version 5
bp: Base pair
CRE: *cis*-regulatory element
FAIRE-Seq: Formaldehyde-Assisted Identification of Regulatory Elements combined with next-generation sequencing
Hi-C: High-throughput chromosome conformation sequencing
lncRNA: Long non-coding RNA
pp: Percentage point
RNA-Seq: RNA sequencing
TSS: Transcription start site
